# Saying it out loud: explicit equity prompts for public health organization resilience

**DOI:** 10.3389/fpubh.2023.1110300

**Published:** 2023-05-19

**Authors:** Margaret Haworth-Brockman, Claire Betker, Yoav Keynan

**Affiliations:** ^1^National Collaborating Centre for Infectious Diseases, Rady Faculty of Health Sciences, University of Manitoba, Winnipeg, MB, Canada; ^2^Department of Community Health Sciences, Max Rady College of Medicine, University of Manitoba, Winnipeg, MB, Canada; ^3^National Collaborating Centre for Determinants of Health, St. Francis Xavier University, Antigonish, NS, Canada; ^4^Department of Medical Microbiology and Infectious Diseases, Max Rady College of Medicine, University of Manitoba, Winnipeg, MB, Canada

**Keywords:** health equity, emergency preparedness, public health, resilience, intervention

## Abstract

**Introduction:**

In the early days of the COVID-19 pandemic there were numerous stories of health equity work being put “on hold” as public health staff were deployed to the many urgent tasks of responding to the emergency. Losing track of health equity work is not new and relates in part to the need to transfer tacit knowledge to explicit articulation of an organization’s commitment to health equity, by encoding the commitment and making it visible and sustainable in policy documents, protocols and processes.

**Methods:**

We adopted a Theory of Change framework to develop training for public health personnel to articulate where and how health equity is or can be embedded in their emergency preparedness processes and documents.

**Results:**

Over four sessions, participants reviewed how well their understanding of disadvantaged populations were represented in emergency preparedness, response and mitigation protocols. Using equity prompts, participants developed a heat map depicting where more work was needed to explicitly involve community partners in a sustained manner. Participants were challenged at times by questions of scope and authority, but it became clear that the explicit health equity prompts facilitated conversations that moved beyond the idea of health equity to something that could be codified and later measured. Over four sessions, participants reviewed how well their understanding of disadvantaged populations were represented in emergency preparedness, response and mitigation protocols. Using equity prompts, participants developed a heat map depicting where more work was needed to explicitly involve community partners in a sustained manner. Participants were challenged at times by questions of scope and authority, but it became clear that the explicit health equity prompts facilitated conversations that moved beyond the idea of health equity to something that could be codified and later measured.

**Discussion:**

Using the indicators and prompts enabled the leadership and staff to articulate what they do and do not know about their community partners, including how to sustain their involvement, and where there was need for action. Saying out loud where there is – and is not – sustained commitment to achieving health equity can help public health organizations move from theory to true preparedness and resilience.

## Introduction

Following the SARS (2003) and H1N1 (2009) pandemics, international (1, 2) and Canadian (3, 4) reviews of public health responses to the emergencies included assessments of how well different populations were served. In Canada, recommendations included calls for an improved focus on at-risk populations (5, 6) and on the interactions and effects of social determinants of health during pandemics, in keeping with a population health approach and commitment to leave no one behind (7). At the national level at least, the reviews determined that certain populations (e.g., Indigenous and northern residents; immigrants from sub-Saharan Africa) were not well served, and in fact suffered unduly because of inconsistent and inequitable public health planning, responses and mitigation (8, 9). Fifteen years later, in a retrospective document, Canada’s Chief Public Health Officer pointed out that there were still gaps in how public health systems address health equity concerns (10).

In other areas of healthcare, ethical and equity analyses have explored how to proportionally allocate resources to those most disadvantaged during the pandemic (11, 12). A US framework, for example, includes equity among its ethical principles: “Aim to offer the same chances of access to indivisible resources to all patients, which does not mean that everyone will receive the same quantity or type of resource. Equity assumes that resources are allocated proportionally to the conditions and needs of individuals, giving priority to the most disadvantaged for reasons that are not only clinical but also socio-economic, since these may be responsible for a worse prognosis” (13).

There are three challenges public health organizations face. The first is how to put knowledge about health equity into practice. The National Collaborating Centres for Determinants of Health (NCCDH) and Infectious Diseases (NCCID) (14) work with public health policy makers and practitioners to highlight the need for health equity in public health programs and services and to prevent and mitigate inequities wherever possible (15). It is our experience that the work of “doing” health equity requires an explicit focus as well as dedicated time and resources. We have developed materials and manuscripts that encourage analyses of performance and health status indicators using disaggregated data and cross-tabulations (16), and also demonstrate explicitly which analyses will help to illuminate health inequalities and health inequities that can be changed (17–19).

The second challenge is how to record the knowledge of and commitment to health equity. Essentially this is a question related to performance measurement. Performance measures, indicators, are used to reflect the intent of an organization to achieve common goals or objectives (such as timely reporting or new strategic partnerships with communities). The measures are used to assess how well an organization is achieving those goals (across years or between departments, for example) and where further action is needed (20). Although performance measurement has been a recognized accountability approach for over 20 years, understanding and using performance measurement indicators is difficult for many people and organizations, particularly if they do not perceive direct relevance to their position and role (21, 22). Incorporating health equity indicators in public health planning and policy is essential, however, even within organizations that have embraced the value of health equity.

A third challenge is related to data systems. These are often separate and do not communicate across health departments and jurisdictions, limiting the ability to document pre-existing inequities. The access to information systems is in itself inequitable, impeding the ability to measure the impact of public health responses. This is especially true among those marginalized in rural and remote settings.

In the early days of the COVID-19 pandemic we heard stories from colleagues in public health authorities (Public Health Units in Ontario, health authorities in different parts of Canada, and several provincial health authorities) of health equity work being put “on hold” as staff were deployed to the many, urgent tasks of responding to the public health emergency. The issue of losing track of health equity work is not new and relates in part to the need to transfer tacit knowledge (often staff members’ personal experience and history) (23) to explicit articulation of an organization’s commitment to health equity. One way to achieve this is to encode the commitment and make it visible and sustainable in policy documents, protocols, and processes.

In this paper, we adopt international definitions of health inequities as health differences between population groups that are created by systematic, avoidable, and unfair structures, such as marginalization (limited or no involvement in decision-making), neglect (dismissal of opportunities and claims to resources that are provided to others), or inadequate (services are not suited for the users) (19). Health equity is thus understood to be the circumstance in which all people can reach their full health potential and are not disadvantaged from attaining it because of their geography, socioeconomic status, social class, ability to find and receive appropriate resources, race, ethnicity, religion, gender, age, sexual orientation or other socially determined circumstance (24). An organization can strive for health equity, fairness, and justice by working to eliminate health differences and impediments to good health that are unnecessary and avoidable (15).

Resilience is defined loosely as an “ability to bounce back.” Organizational resilience denotes an ability of a system to adapt to a challenge or threat, as in the face of emergencies such as pandemics, and to restore a functioning system during the recovery phase (25).

### Training for a public health organization: moving from tacit to explicit

Early in the pandemic, in the spring of 2020, we conducted a scan of national level documents [methods described previously (26)] and reviewed lessons learned about health equity from the SARS and H1N1 epidemics. Two salient papers by Khan et al. describe their research to develop an evidence-informed framework for public health organizational resilience during emergencies and an accompanying set of organizational performance indicators (27, 28).

We built upon the work of Khan et al. and developed health equity prompts for each of the 67 indicators they had identified for the 11 elements in the organizational resilience framework (26). For example, an indicator related to risk assessment for emergency preparedness was originally derived by Khan et al. (28) as, “The public health agency uses the results of the risk assessment to inform relevant plans/protocols for emergency management, business continuity and/or risk reduction.” Our health equity prompt added, *Plans and protocols for emergency management, business continuity or risk reduction are explicit regarding the needs and strengths of disadvantaged populations. For example, they would include older adult women, First Nations, Métis and Inuit communities, families living near hazards*.

For every indicator there is an explicit prompt of how community engagement, documents, human resources, networking, leadership or communications could operationalize a public health organization’s commitment to advancing health equity. Our primary recommendation was for public health organizations to be explicit in their commitment to health equity by specifying that commitment in protocols, policies and processes (26).

The authors made numerous virtual presentations of the framework, indicators and equity prompts to regional, national and federal public health organizations in 2020 and 2021.^1^ When there appeared to be a lull in the COVID-19 pandemic in the summer of 2021, a regional public health organization expressed interest in working with the authors to delve into the practicalities to ensure their commitment to health equity was codified. The organization serves a region of Canada with geographically dispersed populations (total, about 2 million), and north–south and urban–rural disparities in healthcare and public health resources. During emergencies, the organization supports provincial and local response efforts. The purpose of the engagement was to help ensure that in the face of another emergency, efforts for health equity would be sustained. According to Khan et al., this would be a form of resilience within the public health organization itself, to uphold its values and ethics while dealing with a pandemic, natural disaster or other emergency (27).

In this paper we describe training and a follow-up pilot conducted by NCCID and NCCDH with a public health organization to ensure health equity concerns were made explicit. We present the process and outcomes, which highlight the need to say out loud how, and where, to build health equity capacity in organizations as they work to remain resilient during emergencies. The results signify that the training sessions can be used by public health at all levels to ensure contributions and decision-making in emergencies are not done only within power authorities, and contribute to more thoughtful integration of community knowledge in future emergencies.

## Methods

We approached our work within a Theory of Change framework. Theory of Change is a process to comprehensively describe and illustrate how and why a desired change may happen within a specific context. A Theory of Change is “focused in particular on mapping out or “filling in” what has been described as the “missing middle” between what a program or change initiative does (its activities or interventions) and how these lead to desired goals being achieved” (29). As Reinholz and Andrews have noted, “(t)he initial theory of change for a project is really a series of hypotheses about how change will occur” which are explored as the project proceeds. This entails being explicit about the context and circumstances in which the players are operating (30).

The training we conducted built upon a number of foundations: the review of literature done in April 2020 (26); the set of equity prompts written to augment an existing public health organization framework and indicators for organizational resilience during emergencies (26); and presentations made to public health personnel in local, regional and federal authorities and the comments and responses received from medical officers of health, public health planners, program leaders and academics. In addition, the authors drew upon their over 35 years of experience developing, supporting and reinforcing health equity initiatives in public health organizations in Canada and internationally (19, 31–33). The training was informed by a practice framework for organizational capacity for health equity action developed in 2020 in collaboration with several public health experts and organizations (34).

We worked with a four-member leadership team in the public health organization to determine the training objectives, the schedule for training sessions and to encourage consistent participant attendance (i.e., participants would take part in all sessions, confident that they would have protected time to work through the indicators and health equity prompts between sessions).

Materials (agendas, timetables, and assignments) were co-developed with the organization’s leadership team, and it was typical for the authors and the leadership team to meet once or twice to plan before each training session.

A four-part training schedule was developed to support progress through the work. We, the authors, were clear that we were available between sessions to provide coaching or other support as needed.

All sessions started with a review of key concepts (equity, resilience, organizational capacity building), as well as the scope and intent of the project. Session agendas included time for discussion and questions throughout. Virtual breakout rooms and plenary conversations helped to enrich the training experience. In each session, participants were reminded that the equity prompts accompanying the framework and indicators were to be adjusted as needed, according to participants’ roles during emergency preparedness, response and recovery.

Session facilitation was primarily structured using the following questions:

- Which populations are at risk of inequities? (Who is already disadvantaged by the current structures for emergency preparedness, response and recovery?)
- What action is required to mitigate inequities?
- How can that action be taken?
- Who is missing at the decision-making tables, or in the considerations?

Throughout the sessions and meetings, we took field notes of the conversations, issues and challenges. These notes became a record of progress toward the desired change: improved articulation and sustained commitment to health equity in the organization as part of its resilience during an emergency.

Finally, we conducted a short survey on-line with all participants 6 months after the training concluded, asking them to evaluate the training itself and to comment on whether the approach and content has changed their ability to articulate their organization’s commitment to health equity in emergency preparedness and response, within their respective roles. In January 2023, some participants were interviewed by a third party for further follow-up.

## Results

The final training objectives are presented in Table 1 and include: ensure there was consistent understanding and use of terms; allow participants to become familiar with the organizational resilience framework, indicators and the equity prompts; and, determine indicators and prompts as focus for the participants’ detailed, explicit workplan.

**Table 1:** Objectives for organizational training.

Objective	Sub-objective
1. To strengthen understanding of key health equity definitions and concepts	1.1 To understand the core function of public health and the organization’s role to reduce health inequities
2. To understand strategies and methods to systematically integrate health equity considerations into all emergency preparedness and response decisions	2.1 To understand a public health emergency preparedness indicator framework and indicators
	2.2 To understand health equity considerations (prompts) in indicators for organizational resilience
3. To test a framework, indicator, and equity prompts for the organization’s emergency preparedness and response decisions	3.1 To identify the scope of work needed to integrate indicators in all emergency preparedness and response decisions
	3.2 To determine priority indicator areas
	3.3 To begin work of integrating prioritized equity indicators at the organization

### Training agenda

The agenda was designed to be participatory and flexible, to accommodate and respond to the particular issues and populations for the public health organization. Critical to the success of this project was the ability and willingness of the organization staff to spend time between training sessions to review documents, discuss nuances and prepare for the next session. Additionally, we offered to make ourselves available to coach staff and teams as needed.

Fifteen people participated in the training. All sessions were conducted by video conference to comply with COVID-19 restrictions.

The first training session took place in late fall of 2021 and began with a presentation and review of key concepts (equity, equality, resilience, and indicators). We then presented the framework and indicators, with the indicator equity prompts. We reiterated that the prompts were a best draft, and that we expected that the wording would change to suit their organizational structures, the networks and relationships for responding to an emergency, and to reflect the populations they serve and the communities with which they work.

Participants were provided with an Excel worksheet that included the resilience framework, the original 67 indicators and the accompanying explicit prompts for health equity considerations. They were asked to work before the next session in groups to assess their organization’s achievement of select indicators within the framework, assessing whether they considered that their organization met the performance indicator and to color code accordingly (green for achieved, yellow for needing work, and purple for not started). The result was a ‘heat map’ assessment for the indicators. The participants were then asked to apply the equity prompts to the indicators and determine if the explicit health equity prompt changed their assessment of how well they thought they had achieved the performance indicator (and the resultant color coding). No examples were requested for this first exercise, but instead to consider their initial perspectives on how well the organization could document their preparedness and responses to the pandemic.

Between the first and second session, the public health organization completed an assessment (heat map) of all 67 indicators across the 11 elements of the public health organizational framework. The participants worked in small groups on different sections of the spreadsheet and the final compilation was shared with the authors by the leadership team. Interestingly, the training participants had created a fourth color code: blue for “we do not know.” As a group, they had made notes from their discussions about many of the health equity prompts. Table 2 summarizes the results of the heat map across the 11 framework elements.

**Table 2:** Summary of the color-coded heat map generated by the public health organization training participants.

Framework elements (# indicators within element)	With equity prompts
Governance and Leadership (12)	
Planning process (6)	
Risk assessment (5)	
Resources (6)	
Collaborative networks (4)	
Community engagement (4)	
Communication (11)	
Workforce capacity (7)	
Surveillance and monitoring (4)	
Learning and evaluation (3)	
Practice and experience (5)	

Within each element, the majority of indicators were scored as green – “achieved”; yellow – “in process”; purple – “to be addressed”; blue – “we do not know.”

An important finding emerged in this process. Where the staff had assessed the indicators as being achieved (green), or in the process (yellow) of achieving, resilience in emergency preparedness for the organization, when they examined it with the health equity prompts, they found that they scored many of the indicators as, “still to be achieved” (purple). That is, staff found there was not explicit mention of populations, their resilience and knowledge as an asset, or their representation in documents or processes. Whereas, staff said they “knew” where to find the right people and knowledge, it was available to them only as experience, but was not articulated in the organization’s own processes and documents. It would not, for example, be easily shared with someone new to the staff.

In the second training session the full heat map was shared and used to discuss challenges the participants had with conducting the organizational assessment. Primarily, the group was challenged in defining the scope and authority they could own in their replies to the prompts. This required the group to decide on the scope of their individual and organization’s role and reach during an emergency. As one participant noted, “There are so many people at an incident command table, from so many sectors and authority levels. I am not in a position to influence their health equity responses. How far a reach are you expecting us to have?”

The arrival of the Omicron variant of concern (December 2021 to January 2022) delayed the next session by 8 weeks. The third session was used to discuss how the project was proceeding despite the participants having been re-deployed to emergency tasks. Working groups selected a few indicators across the framework to concentrate their efforts and develop action plans. Breakout groups during this session were used to discuss the individual indicators, where the organization’s staff saw gaps, and guidance from the authors about how to address the gaps. In some cases, the staff’s perceived lack of authority proved to be especially challenging.

The fourth, and final, training session provided an opportunity for participants to share their experiences in working through the health equity prompts and their plan as a group to complete the work of articulating the health equity commitments. The organization leadership team committed to finalizing an action plan reflective of the discussion over the previous sessions.

We anticipated that the large number of indicators (and prompts) could be overwhelming. From the outset, we encouraged the organization’s staff to select where they would like to put their emphasis for change. It was beneficial to everyone to have the group score their progress on every indicator and prompt, but it was also understandable that after that initial exercise the leadership encouraged a focus on certain indicators where they saw a need for action and change.

To make that selection, the leadership team considered which indicators were:

- Doable – within scope of the team and organization.
- Important – would emphasize opportunities to ‘embed equity in all that we do’.
- Aligned with a desired outcome – better engagement with the community.
- Aligned with organization priorities – suitable for an ‘after action’ review.
- Aligned with leadership priorities – i.e., intersectoral and cross system collaboration.

Using these criteria, the training participants were able to overcome their initial worry about scope and authority in multi-sector, multi-jurisdictional responses to an emergency.

On the other hand, in the rich discussions it became clear that the explicit health equity prompts moved conversations beyond the idea of health equity, to something that could be codified and later measured. Using the indicators and prompts enabled the leadership and staff to articulate what they do and do not know about their community partners, including how to sustain their involvement, and where there was need for new steps (action). The constant involvement of the leadership team reinforced the value of the training to improve the organization’s commitment to health equity as a core value at all levels.

After the formal training concluded, one author (MHB) received a request from the leadership team to join a working group in a table-top exercise. The scenarios discussed represented the numerous players (sectors and roles) involved in COVID screening at an international airport. Together, we deliberated on how to raise health equity considerations frequently and challenge assumptions team members may have about clients and the services available to them. The success of the direct coaching led to a request for a Self-Reflection tool for the public health organization staff to use in future work.

### Evaluation

Six months after the project concluded, participants evaluated the training sessions to be effective and valuable in increasing their understanding of how well the health equity considerations were already in place in their organization. Twelve participants responded. Ten respondents agreed fully that the training was helpful in “articulating how health equity is demonstrated or achieved.” Furthermore, 11 of the respondents agreed or strongly agreed that the training improved their “understanding of and facility with key health equity concepts.” All but one respondent agreed that the training improved their understanding about strategies and methods to systematically integrate health equity considerations into emergency preparedness and response decisions.

In reply to an open question about how the respondents have or will be using the health equity lens in their work, nine people provided fulsome replies, including:

I was able to see the barriers in addressing health equity at the governance level and hopefully work on creative and collaborative methods to further bridge health equity in EPR [emergency preparedness and response] and potentially on a larger scale. – Respondent 2

From the sessions, I have integrated the information and training into my work processes. The lens has changed to include the health equity pieces as a pillar to the work. – Respondent 4

Initiated conversation with the President, (province) Public Safety Agency who believes this health equity work and tools will complement the Truth and Reconciliation approach their Agency is applying Emergency Management and Response activities. – Respondent 7

A third-party assessment was conducted in January 2023, which consisted of interviewing participants to ask if the training was still relevant 10 months later. Interviewees spoke about having increased confidence to have difficult conversations around equity, an evidence-based framework to guide their work on embedding health equity and tracking progress, and identification of priority focus areas (Table 3).

**Table 3:** Comments from training participants in interviews 10 months after the training.

Interview question	Participant responses
Since the development/use of measuring what counts, what has changed?	“It feels like there is so much work we need to do and sometimes you need something to anchor to. Measuring What Counts allowed us to be like, okay, we do not have to bite off everything. Where do we really need to focus? It’s hard work but it allowed us to narrow in and pinpoint a few actions … it’s a blueprint without being prescriptive.” “{Our organization} has a much stronger commitment to doing this work. It’s hard work. We are still figuring out where we have moved forward in our action plan. We are inching forward. We are moving forward slowly but people see its importance.” “The guidance document gave us language, an evidence-based approach, and the structure to be able to have the conversations around health equity within units that did not fully understand how they could operationally start embedding health equity or see their role in embedding health equity into the work.”
How did NCCID/DH contribute to these changes?” (success factors)	“Part of what made it so successful is that it was joint learning. We were learning together with the National Collaborating Centers. It wasn’t prescriptive … they wanted to learn how this worked in the real world, but they did not have a set idea of exactly how it needed to go. They were willing to work that through with us.” “We were allowed to be the learners. We did not have to be the experts of the work … the NCCs were there to bolster us and support us to do it. I do not think we could have taken it on our own.” “What the NCCss did well was offer up that time and that strong collaboration. We had to work between us too, modelling that collaboration and that common view.”

## Discussion

Since the SARS and H1N1 pandemics, significant attention was put to improving health equity, and public health authorities in Canada took steps to understand local populations and the conditions that influence their health. However, as the COVID-19 pandemic took hold in early 2020, many public health organizations diverted health equity staff and units to disease management tasks. Without information about pre-existing inequities emerging from continuous collection and analysis of disaggregated data by geography, socioeconomic status, race, ethnicity, religion, gender, age, social class, or sexual orientation, designing appropriate public health responses that address inequities is difficult. Furthermore, a lack of systematic, recorded partnerships and protocols meant public health authorities were not able to plan and measure structural performance on integrating health equity in their emergency preparedness planning, response and recovery efforts. The effects of this were amplified as COVID-19 disproportionately affected disadvantaged sub-populations (35, 36).

Although there is considerable scholarship on the theory of health equity and an equally large library devoted to supporting health equity in organizations, it is nonetheless common for public health personnel in agencies (physicians, nurses, epidemiologists, planners) and health authorities (government analysts and policy-makers) to be unsure about how to put the theory of health equity into practice or action (18).

This pilot was specific to the COVID-19 pandemic, but the framework, indicators and equity prompts are designed to be used by public health and related organizations for planning and responses to ongoing epidemics/endemic conditions as well as future emergencies. Explicit prompts are used to sustain attention to equity and community partnerships in performance measurement in other sectors and are considered standards for international reporting, for example (19, 37, 38).

As in other areas of performance measurement, public health organizations need to shift from thinking health equity is a good idea to being able to codify actions that will be taken to prevent or reduce inequities and then continue to track progress on those actions using measurable performance indicators. The pathway to change is created by reflecting explicitly on the current circumstances, where there are gaps, who is missing in the process and how to move towards the desired outcomes (29, 30). Using an organizational assessment tool (the heat map) proved to be a succinct way for participants to quickly assess how well health equity commitments were articulated and to set priorities for action in this pilot. Meeting with the leadership team between the full sessions appears to have helped the entire group understand what was being asked of them and provide their perspectives on the health equity prompts accordingly.

Similar exercises need to be tailored for public health authorities and jurisdictions to provide a platform for increasing awareness and knowledge about health equity and to enhance commitment to health equity. The latter can be attained by incorporating performance measures that reflect the organization mandate and the population it serves and that can drive change towards equitable public health responses.

At the heart of all the equity prompts is an aspiration for public health’s meaningful engagement, partnership and involvement with community. The prompts are not necessarily prescriptive but intended to encourage a range of appropriate equity-informed decisions, actions and process development. Saying out loud where there is – and is not – sustained commitment to achieving health equity can help public health organizations move from theory to true preparedness and resilience.

### Limitations

There were several limitations to this initiative. Using the virtual format meant that we could not be sure that participants were able to be fully engaged in each session, and in fact it was clear at times that some team members were managing other tasks during the sessions.

The participants were managers from different emergency response units, but our sessions did not include representation from external partners representing the communities served, thus the heatmap estimating where the organization is positioned in relation to the equity prompts is biased and broader exercises will improve the accuracy of measures.

The pandemic itself interfered with the flow of the training and some participants expressed frustration with the lack of continuity this created. It is likely that the training content should be adapted to any other public health organization’s circumstances and populations served.

The training and using the heat map were tested for the first time in this pilot. Future training planned with other organizations, will help determine the validity and value of this process. The transferability of lessons learned from this training to other organizations is an area the authors intend to explore.

## Data availability statement

The raw data supporting the conclusions of this article will be made available by the authors, without undue reservation.

## Ethics statement

Ethical review and approval was not required for the study on human participants in accordance with the local legislation and institutional requirements. Written informed consent for participation was not required for this study in accordance with the national legislation and the institutional requirements.

## Author contributions

MH-B, CB, and YK conceptualized the paper. MH-B wrote the initial drafts. CB and YK contributed to revisions and the final manuscript. All authors contributed to the article and approved the submitted version.

## Funding

Production of this document has been made possible through a financial contribution from the Public Health Agency of Canada. The views expressed herein do not necessarily represent the views of the Agency. This is NCCID Project #691.

## Conflict of interest

The authors declare that the research was conducted in the absence of any commercial or financial relationships that could be construed as a potential conflict of interest.

## Publisher’s note

All claims expressed in this article are solely those of the authors and do not necessarily represent those of their affiliated organizations, or those of the publisher, the editors and the reviewers. Any product that may be evaluated in this article, or claim that may be made by its manufacturer, is not guaranteed or endorsed by the publisher.

## Footnotes

^1^See for example: https://nccid.ca/webcast/measuring-what-counts-in-the-midst-of-the-covid-19-pandemic-equity-indicators-for-public-health/?hilite=measuring.
